# Evaluating the diagnostic value of vWF:Ag, D-D and FDP in patients with acute cerebral infarction using ROC curves

**DOI:** 10.3892/etm.2014.1665

**Published:** 2014-04-03

**Authors:** DONGMIN SHI, TING XIA, HONGXUAN FENG, QINGZHANG CHENG

**Affiliations:** 1 Department of Laboratory Medicine, Suzhou Hospital Affiliated to Nanjing Medical University, Suzhou, Jiangsu 215002, P.R. China; 2 First Cadres Sanatorium, Second Military Medical University, Shanghai 200433, P.R. China; 3 Department of Neurology, Suzhou Hospital Affiliated to Nanjing Medical University, Suzhou, Jiangsu 215002, P.R. China

**Keywords:** von Willebrand factor antigen, D-dimer, fibrinogen degradation product, receiver operating characteristic curve, acute cerebral infarction

## Abstract

Cerebral infarction is usually associated with arteriosclerosis, vascular endothelial cell injury and blood flow through the vascular system. The diagnostic value of markers, including von Willebrand factor antigen (vWF:Ag), D-dimer (D-D) and fibrinogen/fibrin degradation product (FDP), have not been studied in patients with acute cerebral infarction. Thus, the aim of the present study was to use receiver operating characteristic (ROC) curves to evaluate the diagnostic significance of vWF:Ag, D-D and FDP in 94 cases of acute cerebral infarction. The results revealed that vWF:Ag and D-D concentrations were significantly higher in acute cerebral infarction patients as compared with the normal controls (P<0.01), whereas no statistically significant difference in FDP was observed between the groups (P>0.01). Plasma vWF:Ag and D-D concentrations significantly correlated with the National Institute of Health Stroke Scale (NIHSS) scores (r=0.625 and 0.582, respectively; P<0.01). In addition, the vWF:Ag concentration significantly correlated with the D-D concentration (r=0.320; P<0.01), whereas FDP concentration did not correlate with D-D or vWF:Ag concentrations or the NIHSS scores (r=0.172, 0.188 and 0.065, respectively; P>0.05). The area under the ROC curve using vWF:Ag as a diagnostic marker in patients with acute cerebral infarction was 0.900, while for D-D the area was 0.795 and for FDP the area was 0.465. Logistic regression analysis revealed that the odds ratios of vWF:Ag and D-D were 16.727 and 2.324, respectively, which were statistically significant (P<0.001 and 0.023, respectively). These results indicated that using vWF:Ag as a diagnostic marker is likely to significantly improve the sensitivity of diagnosing patients with acute cerebral infarction. The diagnostic value of vWF:Ag concentration was significantly higher compared with D-D and FDP levels.

## Introduction

Acute cerebral infarction is ischaemic necrosis or softening of the brain tissue that is caused by blood circulation disorders and ischaemia-hypoxia of the brain. The pathogenesis of acute cerebral infarction is associated with atherosclerosis, vascular endothelial cell injury, artery stenosis and the formation of a primary thrombus. The core of acute cerebral infarction is atherosclerotic occlusion or thrombosis. Vascular endothelial cell injury and platelet activation are involved in thrombogenesis. Von Willebrand factor antigen (vWF:Ag), released by injured vascular endothelial cells, is the key factor in regulating platelet adhesion and promoting thrombus formation. vWF:Ag is regarded as a marker for vascular endothelial cell injury (1). D-dimer (D-D), the final degradation product of cross-linked fibrin, has been demonstrated to have increased concentrations in several acute thrombotic disorders, with high sensitivity and negative predictive values (2). D-D is used as a marker for hypercoagulable states and hyperfibrinolysis, including deep vein thrombosis and pulmonary embolism, with negative predictive values (3). Fibrinogen/fibrin degradation products (FDPs) are protein fragments generated by the action of plasmin on fibrin and fibrinogen and are associated with the activation of fibrinolytic systems. Previous studies have shown that vWF:Ag, D-D and FDP are associated with acute cerebral infarction (4–6), with concentrations being associated with clinical neurological deficits. However, the use of vWF:Ag, D-D and FDP concentrations as risk factors for acute cerebral infarction requires further investigation. Limited clinical assessments with large sample multiangle analyses have been conducted. However, the present study used receiver operating characteristic (ROC) curves of 94 patients with acute cerebral infarction, retrospectively analysing and evaluating the concentrations of vWF:Ag, D-D and FDP with neurological deficits to determine the diagnostic value of the markers for acute cerebral infarction.

## Materials and methods

### Clinical data

A total of 94 cases were selected from patients that had been admitted to the Department of Neurology in Suzhou Municipal Hospital (Suzhou, China) between April 2011 and April 2012. The patients had been diagnosed with acute cerebral infarction within 72 h of onset, as confirmed by computed tomography (CT) or magnetic resonance imaging (MRI) scans. The patients had no history of stroke, mechanical prosthetic valve, warfarin therapy, serious liver and renal dysfunction, inflammation, blood diseases, cancer or autoimmune system diseases. The disease group included 56 males and 38 females aged between 35 and 85 years (average, 68.7±9.8 years). A total of 120 normal age- and gender-matched subjects admitted during the same period were used as the control group. These control subjects included 68 males and 52 females aged between 44 and 83 years (average, 66.3±9.1 years). Prospective participants who had been administered anticoagulant drugs or stypticum were excluded. The study was conducted in accordance with the Declaration of Helsinki and with approval from the Ethics Committee of Suzhou Municipal Hospital. Written informed consent was provided by all the participants.

### Methods

Associations between NIHSS scores and plasma levels of vWF:Ag, D-D (Siemens Healthcare, Erlangen, Germany) and FDP (Sekisui Chemical Co., Ltd., Osaka, Japan) were retrospectively analysed in the two groups. The effect of acute cerebral infarction was evaluated in terms of the levels of consciousness, language, visual-field loss, extraocular movement, ataxia, dysarthria and sensory loss using the NIHSS scores (15 item neurological impairment scale). Ages and genders of the two groups were recorded. Fasting serum was collected using a 109 mmol/l sodium citrate silicon small capacity (2.7 ml) thick double-walled vacuum heparin tube (no dead space). Plasma samples were separated by centrifugation at 1,760 × g for 10 min and the immune scattering turbidimetric method was used to determine the concentrations of vWF:Ag, D-D and FDP.

### Statistical analysis

SPSS 17.0 software (SPSS, Inc., Chicago, IL, USA) was used for statistical analysis. Pearson’s χ^2^ test was used to compare the interclass rate and the results are expressed as the mean ± standard deviation for normal or approximately normal distribution data. Groups were compared using an independent samples t-test and variance analysis, while Pearson’s linear correlation was used for correlation analysis. ROC curves of the results were plotted to calculate the area under the curve and the standard error. Logistic regression was used to analyse independent risk factors. P<0.05 was considered to indicate a statistically significant difference.

## Results

### Clinical data

The mean age did not significantly differ between the two groups (P>0.05). The male to female ratio in the infarction group was 1.47, whereas in the control group, the ratio was 1.31; thus, gender did not significantly differ between the two groups and the χ^2^ test showed no statistically significant differences (χ^2^=0.18 <χ^2^_0.05_; P>0.05). These results indicate that the two groups had comparable ages and gender distributions (Table I).

### vWF:Ag, D-D and FDP level

**Figure 1:**
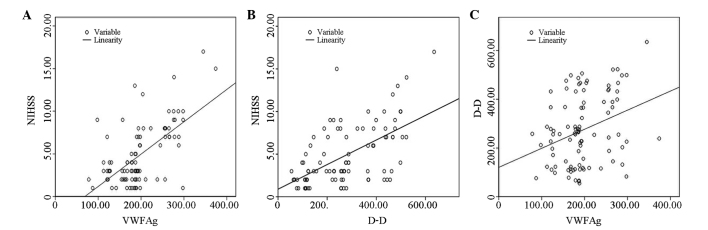
Correlations between (A) vWF:Ag concentration and NIHSS score, (B) D-D concentration and NIHSS score and (C) D-D and vWF:Ag concentrations. vWF:Ag, von Willebrand factor antigen; D-D, D-dimer; FDP, fibrinogen/fibrin degradation product; NIHSS, National Institute of Health Stroke Scale.

vWF:Ag and D-D levels were significantly higher in the acute cerebral infarction patients when compared with the controls (P<0.001), whereas no statistically significant difference in FDP concentration was observed between the groups (P>0.05). The results correlated with the NIHSS scores (Fig. 1).

vWF:Ag and D-D concentrations significantly correlated with the NIHSS scores (r=0.625 and 0.582, respectively; P<0.01). In addition, there was a significant positive correlation between vWF:Ag and D-D concentrations (r=0.320; P<0.01). However, FDP did not correlate with D-D, vWF:Ag or the NIHSS scores (r=0.172, 0.188 and 0.065, respectively; P>0.05) (Fig. 1).

### Comprehensive evaluation of the diagnostic values

Threshold values for vWF:Ag, D-D and FDP in diagnosing acute cerebral infarction were determined using the ROC curves (vWF:Ag, >137%; D-D, >256 μg/l; FDP, >5 mg/l). The sensitivity, specificity and accuracy levels of vWF:Ag were significantly superior compared with D-D and FDP. In addition, the misdiagnosis and missed diagnosis rates of vWF:Ag were lower compared with D-D and FDP (Table II).

### Logistic regression analysis

**Figure 2:**
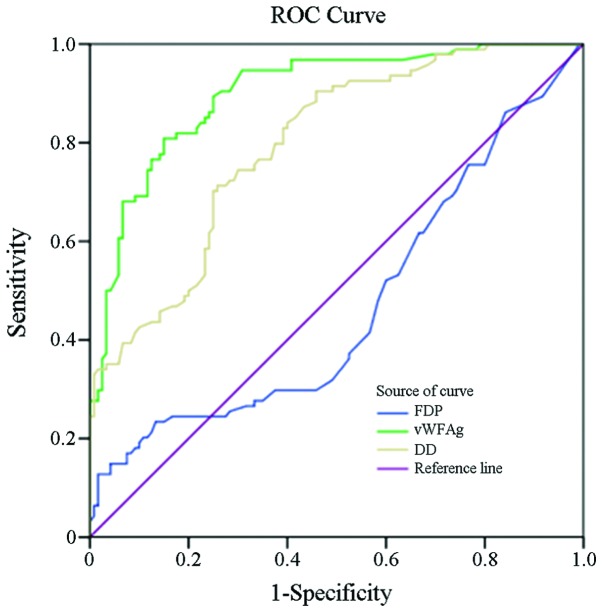
vWF:Ag, D-D and FDP ROC curves. vWF:Ag, von Willebrand factor antigen; D-D, D-dimer; FDP, fibrinogen/fibrin degradation product; ROC, receiver operating characteristic.

Areas under the ROC curve using vWF:Ag, D-D and FDP concentrations as diagnostic markers were 0.900, 0.795 and 0.465, respectively, in patients with acute cerebral infarction (Fig. 2). The 95% confidence intervals of vWF:Ag, D-D and FDP were 0.85–0.94, 0.73–0.85 and 0.38–0.54, respectively (Table III). Therefore, the results demonstrate that the diagnostic value of vWF:Ag was good, however, the diagnostic values of D-D and FDP were generally poor.

Logistic regression analysis revealed that the odds ratio (OR) for vWF:Ag was 16.727 and the OR for D-D was 2.324, which were statistically significant (P<0.001 and 0.023, respectively). Therefore, high levels of vWF:Ag and D-D are risk factors in acute cerebral infarction (P<0.05; Table IV).

## Discussion

Using blood markers is an ideal method for rapidly diagnosing acute cerebral infarction (7). A number of blood markers can be used to predict acute cerebral infarction, although their specificity does not compare with MRI and CT scans. However, the results can complement each other.

vWF:Ag is a glycoprotein that is predominantly synthesised by vascular endothelial cells and megakaryocytes. This glycoprotein is stored in the α-particles of platelets and Weibel-Palade bodies of endothelial cells. Under physiological conditions, vascular endothelial cells exhibit a natural antithrombotic ability, releasing prostacyclin and nitrogen monoxidum to inhibit platelet activation. When vascular endothelial cells are injured, reflex contraction occurs to slow the blood flow and enhance platelet adhesion and aggregation. The factors released by injured endothelial cells are also associated with thrombus formation (8). vWF:Ag is a marker for injury to vascular endothelial cells and the initiating factor for atherosclerosis and thrombosis. A previous study reported that vWF:Ag is one of the risk factors for acute cerebral infarction (9). However, previous studies based on enzyme-linked immunosorbent assay results (10) have shown that the rare large sample multiangle immune turbidimetric method can be used for the emergency care of vWF:Ag-associated acute cerebral infarction (11).

Detecting vWF:Ag, D-D and FDP concentrations using an automatic coagulation analyser by the monoclonal immune turbidimetric method is simple, fast and reliable. Considering vWF:Ag levels are affected by a number of factors, the present study excluded patients with infections and blood diseases. Gender, age and blood collection times of the two groups were recorded. These control measures reduced the effects of confounding factors on the vWF:Ag concentration. The results of the present study demonstrated that vWF:Ag concentrations were significantly higher in cerebral infarction patients as compared with the controls (P<0.01), which is in accordance with the results of previous studies on various populations (12,13). Therefore, vWF:Ag levels reflect vascular endothelial cell injury in patients with acute cerebral infarction.

D-D is the final degradation product of cross-linked fibrin. Levels have been shown to be elevated in several acute thrombotic disorders, with high sensitivity and negative predictive values. D-D reagents from various manufacturers have different normal values. Manufacturers prepare different D-D molecular weight degradation fragments with various monoclonal antibodies, thus, D-D has highly variable weights, ranging between 50 and >228,000 Da (14). In the present study, D-D concentrations were shown to be significantly higher in patients with acute cerebral infarction as compared with the controls (P<0.01). This result indicates that more fibrin is present in the thrombus of acute cerebral infarction patients, thus, D-D levels contribute to the clinical diagnosis of acute cerebral infarction. Studies by Hollestelle *et al* and Chuang *et al* reached the same conclusion (15,16).

FDPs are protein fragments generated by the action of plasmin on fibrin and fibrinogen, and are associated with the activation of fibrinolytic systems, including fibrinogen degradation product, non-cross-linked fibrin and cross-linked fibrin degradation product. Increasing vascular endothelial cell damage and exposure of the subendothelial matrix and collagen fibres causes increased fibrin thrombus formation, activation of the fibrinolytic system, blood hypercoagulability and the incidence of acute cerebral infarction. In the current study, a reference range of <5 mg/l was used. FDP levels reflect fibrinolytic hyperthyroidism. The results demonstrated that the acute cerebral infarction group had higher FDP levels, but no statistical significant difference was observed in FDP between the groups (P>0.05). A recent study by Hirano *et al* (17) revealed that FDP levels were significantly higher in patients with acute cerebral infarction when compared with the controls (P<0.01). However, their results differ to those of the current study. This discrepancy may be due to differences in sample size, detection methods, study design and the use of anticoagulant drugs.

Previous studies have shown that patients with acute cerebral infarction exhibit increasing vWF:Ag levels with the progression of the disease (18). Levels of vWF:Ag and D-D significantly correlated with the NIHSS scores, and vWF:Ag and D-D concentrations also exhibited a significant correlation. However, FDP did not correlate with D-D or vWF:Ag concentrations or the NIHSS scores. Therefore, increasing vascular endothelial cell damage and exposure of the subendothelial matrix and collagen fibres causes increased fibrin thrombus formation, activation of the fibrinolytic system, blood hypercoagulability and the incidence of acute cerebral infarction.

The area under the ROC curve is widely used for measuring the performance of classification and diagnostic criteria. Theoretically, the area under the curve is 0.5≤AUC≤1, with higher areas indicating increasing diagnostic values. In the present study, the area under the ROC curve using vWF:Ag as a diagnostic marker for acute cerebral infarction was 0.900, while for D-D and FDP the areas were 0.795 and 0.465, respectively. These observations indicate that the accuracy of the markers for diagnosing acute cerebral infarction is in the following order: vWF:Ag>D-D>FDP. Thus, the diagnostic value of vWF:Ag was satisfactory, but better compared with D-D.

Logistic regression was used to analyse the significance of independent risk factors. The results demonstrated that high levels of vWF:Ag and D-D were risk factors for acute cerebral infarction. In addition, logistic regression analysis revealed that the ORs of vWF:Ag and D-D were highly significant. Thus, vWF:Ag and D-D are risk factors for acute cerebral infarction.

In conclusion, the present study firstly demonstrated that vWF:Ag and D-D levels linearly correlate with NIHSS scores. Secondly, diagnosing acute cerebral infarction using vWF:Ag via immune turbidimetry is more accurate than using D-D concentration, however, D-D is more accurate compared with FDP. Thirdly, vWF:Ag has a high negative predictive value and can be used as a diagnosis marker for acute cerebral infarction. The sensitivity and specificity values for diagnosing acute cerebral infarction with vWF:Ag were 84% and 78%, respectively. Finally, vWF:Ag levels are a risk factor for acute cerebral infarction. Atherosclerosis occlusion and thrombosis are associated with acute cerebral infarction, vascular endothelial cell injury and platelet activation. Early disease detection of acute cerebral infarction using plasma vWF:Ag and D-D levels can protect vascular endothelial cells from further damage and improve the level of treatment and patient prognosis.

## Figures and Tables

**Table I:** Levels of vWF:Ag, D-D and FDP in patients with acute cerebral infarction.

Groups	Cases, n	vWF:Ag, %	D-D, μg/l DDU	FDP, mg/l
Disease	94	196.73±58.19	274.38±142.43	4.53±9.20
Control	120	111.19±40.43	133.05±94.38	2.58±3.36
P-values		<0.001	<0.001	0.054

vWF:Ag, von Willebrand factor antigen; D-D, D-dimer; FDP, fibrinogen/fibrin degradation product.

**Table II:** Diagnostic value of vWF:Ag, D-D and FDP for acute cerebral infarction.

Test variables	Sensitivity, %	Specificity, %	Accuracy, %	Misdiagnosis rate, %	Missed diagnosis rate, %
vWF:Ag >137%	84	78	80	22	16
D-D >256 μg/l	51	76	65	24	49
FDP >5 mg/l	19	90	58	10	81

vWF:Ag, von Willebrand factor antigen; D-D, D-dimer; FDP, fibrinogen/fibrin degradation product.

**Table III:** Area under the ROC curve, standard error, P-values and 95% CI for the test variables.

Test variables	Area under the curve	Standard error	P-values	95% CI
vWF:Ag	0.900	0.021	<0.001	0.858–0.941
D-D	0.795	0.030	<0.001	0.736–0.853
FDP	0.469	0.041	0.438	0.389–0.549

vWF:Ag, von Willebrand factor antigen; D-D, D-dimer; FDP, fibrinogen/fibrin degradation product; ROC, receiver operating characteristic; CI, confidence interval.

**Table IV:** Logistic regression analysis of the risk factors.

Variable	Regression coefficients	Standard error	Wald χ^2^	P-values	OR	95% CI
Constant	−2.174	0.322	45.726	<0.001	0.114	
vWF:Ag	2.817	0.365	59.435	<0.001	16.727	8.173–34.232
D-D	0.843	0.371	5.156	0.023	2.324	1.122–4.811
FDP	0.712	0.518	1.888	0.169	2.039	0.738–5.633

vWF:Ag, von Willebrand factor antigen; D-D, D-dimer; FDP, fibrinogen/fibrin degradation product; CI, confidence interval; OR, odds ratio.
